# Hypoxia promotes stem cell-like phenotype in multiple myeloma cells

**DOI:** 10.1038/bcj.2014.82

**Published:** 2014-12-05

**Authors:** B Muz, P de la Puente, F Azab, M Luderer, A K Azab

**Affiliations:** 1 Department of Radiation Oncology, Cancer Biology Division, Washington University in Saint Louis School of Medicine, Saint Louis, MO, USA

Multiple myeloma (MM) is a plasma cell malignancy affecting the bone marrow (BM); despite the introduction of novel therapies, >90% of the MM patients relapse owing to drug resistance and microresidual disease.^1^ Relapse and microresidual disease in MM may be attributed to the development of a stem cell-like subpopulation, which demonstrates resistance to therapy and causes recurrence.^2, 3^

In solid tumors, stem cell-like cells were found to be responsible for tumor recurrence.^4^ Cancer stem cells are generally identified by (i) the expression of common stem cell and early-differentiation markers, (ii) G1-arrest and quiescent phenotype, (iii) capability of fast tumor initiation, (iv) chemoresistance, (v) high expression of drug transporters and enzymes detoxifying drugs; and (vi) acquisition of the epithelial-to-mesenchymal transition (EMT)-phenotype.^4, 5^ The stem cell-like cells were shown to adapt to hypoxic conditions to promote undifferentiated and immature phenotype of cells,^6^ which was associated with hypoxia-induced activation of stem cell related genes and pathways, such as Oct4, HIF, Notch, Wnt and Hedgehog.^7^

Similar to solid tumors, a subpopulation of MM cells was shown to demonstrate cancer stem-cell-like properties by showing clonotypic properties,^3^ drug resistance and high drug efflux capacity,^3^ and EMT-like phenotype.^8^ Tumor hypoxia was shown to develop during MM progression and to be crucial for metastasis.^8, 9^ Hypoxia was previously shown to induce the acquisition of EMT-like phenotype;^8^ and to promote less mature phenotype by the downregulation of plasma cell factors (IRF4, PRDM1 and XBP1) and upregulation of B-cell- (BCL6 and PAX5) and stem cell transcription factors (Oct4, NANOG, SOX2).^10^ CD138 (syndecan-1, a heparin sulfate proteoglycan) is the gold standard for diagnosis;^11^ however, previous studies showed that MM contains a subpopulation of cells that are CD138-negative, clonotypic, drug resistant, and show stem cell-like properties, express drug efflux pump,[Bibr bib12]^12^ and display higher clonogenic potential than CD138+ *in vivo*.^13^ However, the molecular mechanisms leading to the development of this CD138-negative population and the activation of the stem-cell signaling pathways in them is not yet understood.

In this study, we investigated the effect of hypoxia on the acquisition of stem cell-like properties such as early-differentiation markers, G1-arrest, quiescent phenotype, capability of tumor initiation *in vivo* and drug resistance in MM cells.

To investigate the effect of hypoxia on the differentiation status of MM cells, five MM cell lines were cultured under normoxic (21% O_2_) and hypoxic conditions (in hypoxic chamber; 1% O_2_) for 48 h, and the expression of plasma cell marker (CD138), B-cell markers (CD19, CD20 and CD45) and hematopoietic stem cell marker (CD34) were tested. Consistently with previous studies,^10^ it was found that hypoxic MM cells expressed ~50% less plasma cell marker CD138 compared with normoxic conditions. On the contrary, B-cell markers such as CD20 and CD45 were increased by hypoxia, with no effect on CD19 expression. Moreover, stem cell marker CD34 was increased during hypoxia in MM cells (Figure 1a). These results confirmed that hypoxia induces immature phenotype in myeloma cells in which it decreased the expression of terminal differentiation markers (CD138) and increased the expression of B-cell and stem cell markers.

We further analyzed the effect of hypoxia on cell proliferation and cell cycle in MM cells. MM cells were cultured in hypoxic and normoxic conditions, and their proliferation and associated cell signaling was analyzed. Hypoxia decreased the proliferation of MM cells by 10–20% (Figure 1b), which was associated with decreased activation of the PI3K signaling (p-PI3K-P85, p-AKT and p-mTOR), whereas stress kinases such as p-MKK3/6 and p-P38 were increased in hypoxia (Figure 1c). The reduced proliferation in hypoxia was facilitated through a G1-cell cycle arrest, with decreased synthesis of DNA (S phase) in all three cell lines (Figure 1d), which was associated with the downregulation in the expression of proteins involved in cell cycle transition from G1 to S phase, including cyclin-D1, cyclin-D2, cyclin-D3, cyclin-E and pRb, whereas the cell cycle inhibitor p27 was increased (Figure 1e). In addition, we tested the effect of hypoxia on cell apoptosis and found that both normoxic and hypoxic cells had similar percentage of apoptotic (APO 2.7-positive) cells (Figure 1f). Unchanged apoptosis was confirmed by immunoblotting showing a lack of induction of caspase-3 and caspase-9 cleavage, and unchanged expression of pro- and antiapoptotic proteins such as Bcl-2, Bcl-xL and Mcl-1 (Figure 1g). These results suggest that hypoxia induces a quiescent state of cancer cells with no signs of cell apoptosis.

Moreover, we tested the effect of hypoxia on the ability of MM1s-GFP-Luc cells to initiate tumor *in vivo*. MM cells were incubated in hypoxia or normoxia for 24 h *in vitro* and these were injected via tail vein to SCID (severe combined immunodeficiency) mice, which were followed by bioluminescence imaging for tumor progression. It was found that hypoxia enhanced the tumor initiation ability of MM cells, in which at week 3 the signal appeared first in the mice injected with hypoxic cells but not in animals injected with normoxic cells. Moreover, over the next 2 weeks the mice injected with hypoxic cells demonstrated higher tumor burden (Figures 2a and b). These findings can be attributed to the more efficient homing of hypoxic cells to the BM, as we previously described.^8^ Similar results were previously obtained when stem cell-like MM cells (identified as CD138-/CD34+) induced MM tumors *in vivo* faster and to a higher tumor burden compared with CD138+/CD34-MM cells.^3^

Another important feature of stem cell-like cancer cells is drug resistance. We investigated the effect of hypoxia on MM cell proliferation in the presence of proteasome inhibitors. MM cells were cultured for 24 h in hypoxic and normoxic conditions, and subsequently treated with or without bortezomib and carfilzomib, and the proliferation was analyzed by MTT (3-(4,5-dimethylthiazol-2-Yl)-2,5-diphenyltetrazolium bromide) assay. In normoxia, bortezomib (5 nm) and carfilzomib (5 nm) exerted about 50% of killing (IC_50_) in tested cell lines. However, hypoxia induced complete resistance to the same concentrations of both bortezomib and carfilzomib in OPM1 cells, whereas in the case of MM1s and H929 hypoxia induced partial resistance to both drugs (Figure 2c). It has been demonstrated recently that proteasome inhibitor MG-132, led to increased reactive oxygen species (ROS) production as a potential mechanism for the induction of apoptosis in tumor cells.^14^ Hypoxia was shown to decrease ROS production;^15, 16, 17^ therefore we suggest that this could be a potential mechanism of resistance to proteasome inhibitors in hypoxia in MM cells. In general, these results on stem cell drug resistance are in accord with previous reports showing that myeloma stem cell-like cells (defined as CD138-CD19+CD27+) had an increased activity of aldehyde dehydrogenase (ALDH1), and increased the expression of drug efflux pumps such as ABCG2, ALDH1 and RARα2, conferring chemoresistance to bortezomib, dexamethasone and lenalidomide.^3^ Moreover, the CD138-negative subpopulation of 5T33MM and 5TGM1 cells was also more resistant to melphalan and lenalidomide than CD138+ cells.^18^

The hypoxic phenotype of MM cells was shown to be reversible after reoxygenation. We have previously demonstrated that hypoxic MM cells recovered, as soon as, 6 h after the exposure to medium from normoxic stroma.^8^ In addition, we have recently demonstrated that reoxygenation of hypoxic Waldenström's macroglobulinemia cells reversed their hypoxic phenotype by restoring cell proliferation rate, inducing the exit of the G1-arrest, and restoring E-cadherin expression and adhesion to stromal cells.^19^ Moreover, the expression of the main plasma cell marker CD138, which is decreased by hypoxia, also recovered after reoxygenation in MM cells.^10^

In summary, hypoxia induced MM cell dedifferentiation; acquisition of a quiescent state by decreasing proliferation and inducing G1-cell cycle arrest, but without altering apoptosis; enhanced tumor initiation; and increased drug resistance to proteasome inhibitors. On the basis of these findings we propose to target hypoxic cells to diminish the stem cell-like population, in order to decrease microresidual disease and ultimately prevent recurrence in MM patients.

## Figures and Tables

**Figure 1:**
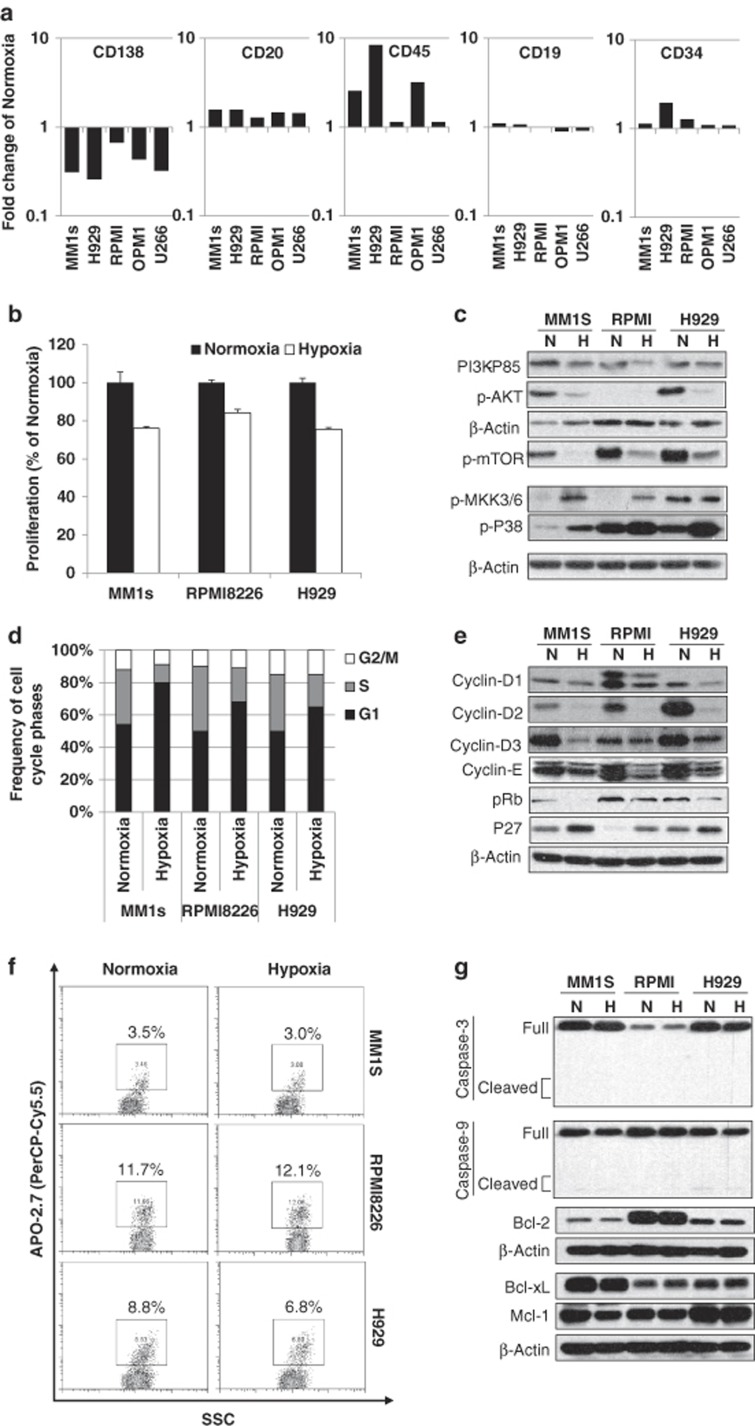
Hypoxia promotes dedifferentiation, decreases cell proliferation, induces G1-cell cycle arrest, but not apoptosis of MM cells. The effect of hypoxia (1% O_2_) on (**a**) CD138-PerCP-Cy5.5, CD20-PE, CD45-APC, CD19-FITC and CD34-PE expression, analyzed by flow cytometry and shown as fold change of normoxia in MM1s, H929, RPMI8226, OPM1, U266 cell lines; (**b**) proliferation of MM cells analyzed by MTT (3-(4,5-dimethylthiazol-2-Yl)-2,5-diphenyltetrazolium bromide) assay; (**c**) the expression of PI3K signaling pathway and stress kinases by immunoblotting; (**d**) cell cycle analysis tested by propidium iodide staining and flow cytometry and shown as the percentage of cells in G1, S and G2/M phases; (**e**) the expression of cell cycle-related proteins detected by immunoblotting; (**f**) cell apoptosis performed using APO 2.7 (PerCP-Cy5.5) staining and shown as the percentage of apoptotic (APO 2.7-positive) cells; and (**g**) apoptosis-related proteins detected by immunoblotting.

**Figure 2:**
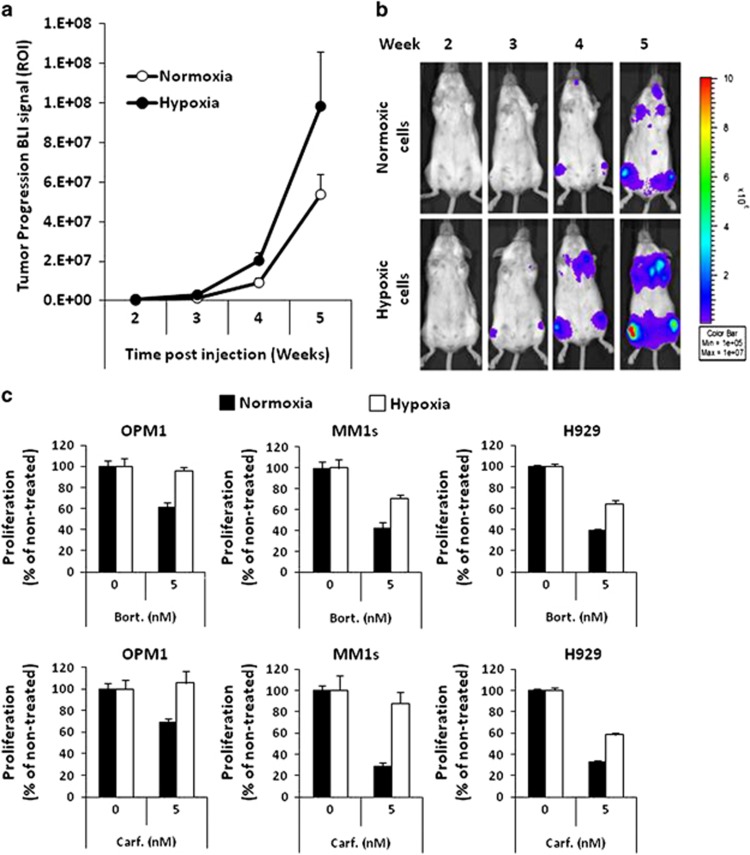
Hypoxia induces rapid tumor initiation and drug resistance to bortezomib and carfilzomib in MM cells. MM1s-Luc-GFP cells were incubated in normoxia or hypoxia for 24 h and injected intravenously into three severe combined immunodeficiency (SCID) mice per condition at the concentration of 2 × 10^6^ cells per mouse. The effect of hypoxia (1% O_2_) on tumor initiation and tumor progression in SCID mice was monitored for 5 weeks using bioluminescent imagining (BLI) and shown as the average of the luminescent signal of three mice (**a**), and as representative BLI pictures of mice injected with normoxic or hypoxic cells at weeks 2, 3, 4 and 5 (**b**). The effect of hypoxia on drug resistance in MM cells shown as the proliferation of MM cells after the treatment with 5 nm bortezomib or 5 nm carfilzomib, analyzed using MTT (3-(4,5-dimethylthiazol-2-Yl)-2,5-diphenyltetrazolium bromide) assay (**c**).
